# *Helicobacter Pylori*‐Enhanced hnRNPA2B1 Coordinates with PABPC1 to Promote Non‐m^6^A Translation and Gastric Cancer Progression

**DOI:** 10.1002/advs.202309712

**Published:** 2024-06-17

**Authors:** Yi Yu, Yan‐Ling Yang, Xiao‐Yu Chen, Zhao‐Yu Chen, Jin‐Shui Zhu, Jing Zhang

**Affiliations:** ^1^ Department of Gastroenterology Shanghai Sixth People's Hospital Affiliated to Shanghai Jiao Tong University School of Medicine Shanghai 200233 China

**Keywords:** gastric cancer, *Helicobacter pylori*, hnRNPA2B1, non‐m6A, translation

## Abstract

*Helicobacter pylori (H. pylori)* infection is the primary risk factor for the pathogenesis of gastric cancer (GC). N6‐methyladenosine (m^6^A) plays pivotal roles in mRNA metabolism and hnRNPA2B1 as an m^6^A reader is shown to exert m^6^A‐dependent mRNA stabilization in cancer. This study aims to explore the role of hnRNPA2B1 in *H. pylori*‐associated GC and its novel molecular mechanism. Multiple datasets and tissue microarray are utilized for assessing hnRNPA2B1 expression in response to *H. pylori* infection and its clinical prognosis in patients with GC. The roles of hnRNPA2B1 are investigated through a variety of techniques including glucose metabolism analysis, m^6^A‐epitranscriptomic microarray, Ribo‐seq, polysome profiling, RIP‐seq. In addition, hnRNPA2B1 interaction with poly(A) binding protein cytoplasmic 1 (PABPC1) is validated using mass spectrometry and co‐IP. These results show that hnRNPA2B1 is upregulated in GC and correlated with poor prognosis. *H. pylori* infection induces hnRNPA2B1 upregulation through recruiting NF‐κB to its promoter. Intriguingly, cytoplasm‐anchored hnRNPA2B1 coordinated PABPC1 to stabilize its relationship with cap‐binding eIF4F complex, which facilitated the translation of CIP2A, DLAT and GPX1 independent of m^6^A modification. In summary, hnRNPA2B1 facilitates the non‐m^6^A translation of epigenetic mRNAs in GC progression by interacting with PABPC1‐eIF4F complex and predicts poor prognosis for patients with GC.

## Graphical abstract

*Helicobacter pylori (H. pylori)* infection is a key risk factor for gastric cancer (GC). This study explores hnRNPA2B1's role in *H. pylori*‐associated GC. hnRNPA2B1, an m^6^A reader, is found to be upregulated in GC and linked to poor prognosis. *H. pylori*‐induced hnRNPA2B1 via NF‐κB. Silencing hnRNPA2B1 reduced glycolysis, tumor growth, metastasis, and increased cisplatin sensitivity. hnRNPA2B1 binds non‐m^6^A mRNAs, coordinating with PABPC1 and eIF4F to facilitate the translation of CIP2A, DLAT, and GPX1, indicating a novel mechanism in GC progression.

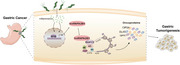

## 1 Introduction

Gastric cancer (GC) is the fifth most common cancer and the third leading cause of cancer‐related deaths worldwide, posing threat to human health.^[^ ^1^, ^2^ ^]^ Although multiple strategies have been used for the treatment of GC such as surgical resection, radiotherapy and chemotherapy, immunotherapy, targeted therapy, etc,^[^ ^3^ ^]^ the advanced patients with GC still harbor poor prognosis ascribed to tumor invasiveness and metastasis. Therefore, it is essential to unravel the molecular pathogenesis of GC and explore the personalized treatment for GC.

N6‐methyladenosine (m^6^A) is the most prevalent modification in mammalian messenger RNAs (mRNAs).^[^ ^4^, ^5^ ^]^ The dysregulation of m^6^A regulators, including writers (METTL3, METTL14, WTAP), erasers (FTO, ALKBH5), and readers (YTHDF proteins, YTHDC proteins, IGF2BP Family, hnRNPC and hnRNPA2B1), disrupts normal RNA processing, stability, and translation.^[^ ^6^, ^7^ ^]^ Multiple “‘readers”’ have been reported to guide protein synthesis through recognizing and binding to m^6^A‐modified transcripts, of which YTHDF1 and YTHDF3 enhance translation initiation of m^6^A‐modified transcripts,^[^ ^8^, ^9^ ^]^ YTHDF2 promotes the degradation of m^6^A‐modified mRNAs, leading to the mRNA instability^[^ ^10^ ^]^ and IGF2BPs facilitate mRNA stability and translation^[^ ^11^ ^]^ Moreover, hnRNPA2B1 can engage in various stages of RNA synthesis and processing. For example, hnRNPA2B1 recognizes and binds to the transcripts compassing pri‐miRNAs, reinforcing DGCR8's affinity for these transcripts and their maturation^[^ ^12^ ^]^ hnRNPA2B1 also regulates mRNA splicing efficiency and mRNA stability potentially through its interaction with m^6^A‐modified pre‐mRNAs^[^ ^13^ ^]^ despite these, how hnRNPA2B1 determines RNA fate in GC remains largely unknown.

*H. pylori* as one of the most prevalent bacteria constructs a predominant risk factor, contributing to gastric mucosal inflammation and cancer transformation^[^ ^14^ ^]^ It is known that *H. pylori* can activate various signaling pathways including NF‐κB and accelerate uncontrolled GC cell growth and metastasis.^[^ ^15^, ^16^ ^]^ However, whether *H. pylori* mediates hnRNPA2B1 to lead to GC remains elusive.

The mRNA translation is a vital biological process that underpins all cellular activities and functions^[^ ^17^ ^]^ Dysregulated mRNA translation results in overproduction of specific proteins and cancer progression^[^ ^18^ ^]^ The initiation of mRNA translation involves recognizing mRNA's 5′ cap, forming the eIF4F complex including eIF4E, eIF4A, and eIF4G on the ribosome, and accurately selecting the start codon^[^ ^19^ ^]^ This step ensures the translation fidelity by integrating multiple cellular signals^[^ ^19^ ^]^ Moreover, poly(A) binding protein cytoplasmic 1 (PABPC1) has been reported to interplay with the eIF4F complex to facilitate mRNA circularization and cap‐dependent translation, stabilize mRNAs, and integrate regulatory signals^[^ ^20^ ^]^ These translation processes are required for the accurate and efficient synthesis of proteins implicated in cancer cellular growth and survival.^[^ ^21^, ^22^ ^]^ In this study, our findings unveil a novel mechanism by which *H. pylori*‐enhanced hnRNPA2B1 coordinates with PABPC1 to promote non‐m^6^A translation and gastric tumorigenesis. These findings indicate hnRNPA2B1 as a potential prognostic factor and molecular vulnerability in GC.

## 2 Results

### 2.1 hnRNPA2B1 is Upregulated and Associated with Poor Prognosis in Patients with GC

**Figure 1:**
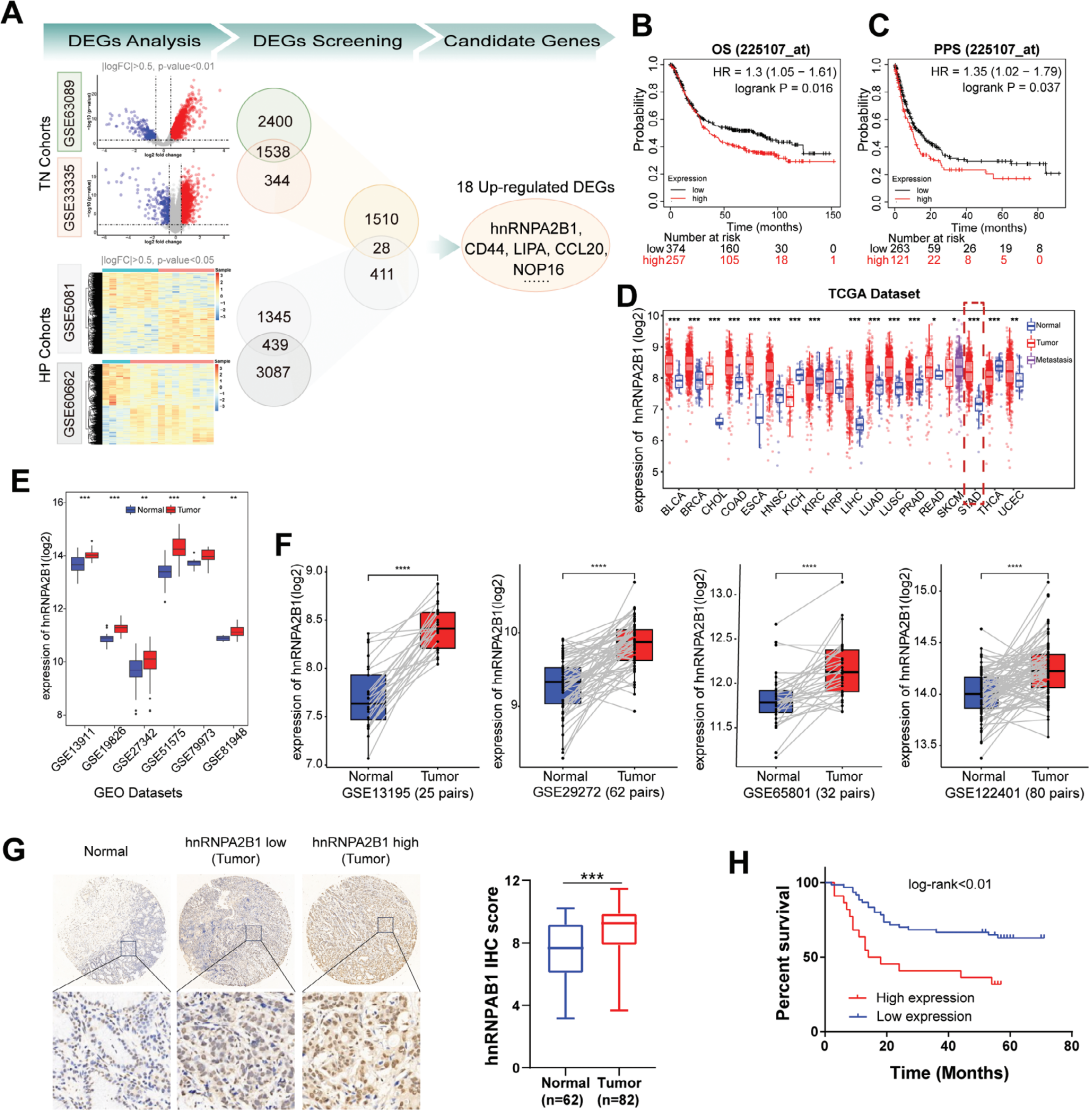
The expression and clinical prognosis of hnRNPA2B1 in patients with GC. A) Analyses of each 2 GEO datasets from tumor versus normal (TN) and *H. pylori* (*HP*) cohorts. Hierarchical clustering heatmap and volcano plots of differentially expressed genes (DEGs) between TN and *HP c*ohorts. Overlapping TN cohorts and HP cohorts initially identified 28 DEGs including 18 up‐regulated DEGs. B,C) Kaplan‐Meier plotter analysis of the association of hnRNPA2B1 expression with overall survival (OS, B) and post‐progression survival (PPS, C) in patients with GC, respectively. D) Analyses of TCGA pan‐cancer database displayed diverse mRNA expression patterns of hnRNPA2B1 across multiple cancer types. E) Analyses of multiple public GEO datasets showed the increased mRNA levels of hnRNPA2B1 in GC samples. F) GEO datasets showed the mRNA expression levels of hnRNPA2B1 in paired tumor and adjacent normal tissues. G) Representative IHC staining of hnRNPA2B1 in human GC and normal tissues from TMA analysis, and the histogram depicting the distribution of hnRNPA2B1 staining scores in TMA cohort. H) Kaplan‐Meier survival analysis of the association of hnRNPA2B1 with OS in GC patients from TMA cohort. ^*^ *p* < .05, ^**^ *p* < .01, ^***^ *p* < .001, ^****^ *p* < .0001.

In our comprehensive analysis utilizing the Gene Expression Omnibus (GEO) public database, we aimed to identify key genes exhibiting irregular expression associated with gastric carcinogenesis, with a particular focus on genes closely associated with *H. pylori* infection. Notably, we designated the GSE63089 and GSE33335 datasets as tumor‐versus‐normal (TN) cohorts, while the GSE5081 and GSE60662 datasets were categorized as *H. pylori* infection cohorts. Our analysis yielded 1538 and 439 significantly differentially expressed genes (DEGs) within the TN and *H. pylori* cohorts, respectively, meeting stringent significance criteria (|logFC| > 0.5 and *p* < 0.01 for TN cohorts; |logFC| > 0.5 and *p* < 0.05 for *H. pylori* cohorts, **Figure** **1**A). By intersecting the DEGs from both TN and *H. pylori* cohorts, we identified a final set of 28 DEGs, encompassing 18 genes, including hnRNPA2B1, which demonstrated significant upregulation in GC (Figure 1A). These upregulated DEGs emerged as pivotal candidates exhibiting aberrant expression in human gastric tumors, potentially contributing to *H. pylori* infection‐driven gastric carcinogenesis. Within this subset of 18 genes, our particular focus was honed on hnRNPA2B1 due to compelling evidence. Utilizing the Kaplan–Meier plotter platform, we ascertained that elevated hnRNPA2B1 expression was correlated with diminished overall survival (OS; P = 0.016, Figure 1B) and post‐progression survival (PPS; P = 0.037, Figure 1C), thereby establishing its adverse prognostic significance in patients with GC. We observed a pronounced upregulation of hnRNPA2B1 in various TCGA‐gastrointestinal tumor datasets, encompassing esophageal carcinoma (ESCA, *p* < 0.001), stomach adenocarcinoma (STAD, *p* < 0.001), colon adenocarcinoma (COAD, *p* < 0.001), and rectum adenocarcinoma (READ, *p* < 0.05) (Figure 1D). This conspicuous elevation in gene expression underscored the prominence of hnRNPA2B1 in gastrointestinal malignancies. The robustness of this finding validated across diverse datasets and strengthens our confidence in the pivotal role of hnRNPA2B1 in gastrointestinal malignancies. Subsequently, we corroborated the elevated expression of hnRNPA2B1 in GC through extensive analysis of several additional databases (all *p* < 0.05, Figure 1E). Also, our findings were substantiated by comparing tumor samples with paired adjacent normal samples from GEO datasets (GSE13195, GSE29272, GSE65081, and GSE122401) (all *p* < 0.0001, Figure 1F).

We further validated the hnRNPA2B1 expression status and their clinical relevance using our own clinical GC tissue cohort. Quantitative analysis revealed a marked upregulation of hnRNPA2B1 in GC samples compared to normal gastric tissues (*p* < 0.001, Figure 1G). Consistent with the above results, Kaplan‐Meier survival curves showed that patients with high hnRNPA2B1 expression levels harbored pfoorer OS (*p* < 0.01, Figure 1H). Hence, this compelling evidence directed our focus toward the potential functions of hnRNPA2B1 in GC.

### 2.2 *H. Pylori*‐Induced NF‐κB Recruitment to the hnRNPA2B1 Promoter Increases its Expression

**Figure 2:**
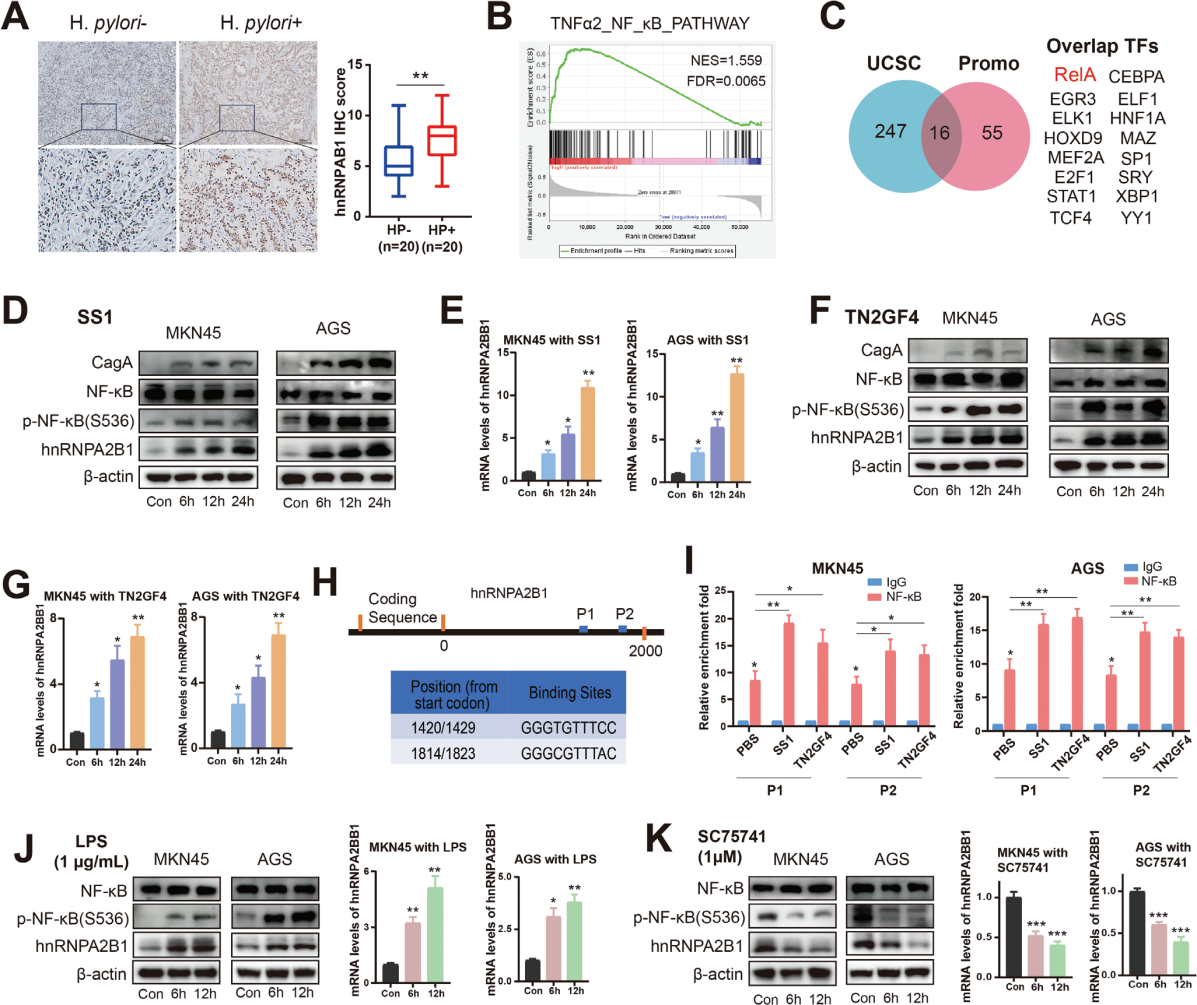
*H. pylori* infection enhanced the transcriptional expression of hnRNPA2B1 via recruiting NF‐κB. A) Representative IHC staining and scores of hnRNPA2B1 in *H. pylori*‐negative (HP−; n = 20) and *H. pylori*‐positive (HP+; n = 20) GC patients. B) GSEA analysis of the downstream signaling of *H. pylori* infection, such as NF‐κB signaling according to hnRNPA2B1 expression in TCGA dataset. NES, normalization enrichment score; FDR, false discovery rate. C) The transcription factor binding sites were predicted by the UCSC and PROMO websites using a 2000‐bp conserved segment of the hnRNPA2B1 promoter. D,E) Western blot and RT‐qPCR analysis in MKN45 and AGS cells following *H. pylori* SS1 infection; F,G) Western blot and RT‐qPCR analysis in MKN45 and AGS cells following *H. pylori* TN2GF4 infection; (H) A scheme and a table showing 2 putative NF‐κB transcription factor binding sites on the hnRNPA2B1 promoter (P1 and P2). I) CUT&Tag assays using NF‐κB antibody were performed and quantified by RT‐qPCR on primers covering P1 and p2 region. J,K) Western blots and RT‐qPCR analysis in MKN45 and AGS cells treated with LPS (J) or SC75741 (K). ^*^ *p* < .05, ^**^ *p* < .01, ^***^ *p*< .001.

In the light of our hypothesis that hnRNPA2B1 might play a pivotal role in *H. pylori*‐associated GC, we aimed to gain a comprehensive understanding of how *H. pylori*‐hnRNPA2B1 interaction contributes to gastric carcinogenesis. First, we analyzed hnRNPA2B1 expression in GC samples stratified with *H. pylori* positive and negative status. Our findings indicated a remarkable upregulation of hnRNPA2B1 in GC with *H. pylori* infection (**Figure** **2**A). The NF‐κB signaling can be commonly activated following *H. pylori* infection. Utilizing GSEA, we corroborated that the cohort associated with elevated hnRNPA2B1 expression, based on TCGA database displayed increased activities in the NF‐κB signaling pathway (Figure 2B). To precisely predict the transcription factors (TFs) that can bind to the hnRNPA2B1 promoter, we conducted UCSC (https://genome.ucsc.edu/) and promo web site (https://alggen.lsi.upc.es/cgibin/promo_v3/promo/promoinit.cgi?dirDB=TF_8.3) to identify 13 overlapping TFs, among which the RELA transcription factor related to NF‐κB pathway exhibited an elevated JASPAR score (http://jaspar.genereg.net/), highlighting its potential in the transcriptional regulation network associated with hnRNPA2B1 (Figure 2C). Western blot analysis showed that *H. pylori* strain SS1 (Figure 2D) and TN2GF4 (Figure 2F) infection raised hnRNPA2B1 protein levels and induced an increase of p‐P65 (S536) in GC cells. Consistently, RT‐qPCR analysis corroborated the induction of hnRNPA2B1 mRNA overexpression upon *H. pylori* strain SS1 (Figure 2E) and TN2GF4 (Figure 2G). These findings indicated that hnRNPA2B1 upregulation was mediated by transcriptional regulation mechanism.

We focused on the 2 putative NF‐κB (RELA) binding regions (P1 and P2) with higher JASPAR scores indicative of binding affinity (Figure 2H). Using the Cut&Tag assay, we observed the recruitment of p65 to the hnRNPA2B1 promoter regions induced by *H. pylori* infection compared with the control groups (Figure 2I). To elucidate the regulatory effect of NF‐κB on hnRNPA2B1 transcription, we further verified these findings by using LPS, a known activator of NF‐κB, and SC75741, a recognized NF‐κB inhibitor. Western blot and RT‐qPCR analyses showed that LPS enhanced the transcriptional levels of hnRNPA2B1 (Figure 2J) but SC75741 effectively suppressed its expression (Figure 2K), emphasizing the central role of NF‐κB in modulating hnRNPA2B1 transcription. These diverse data illustrated that *H. pylori* infection induced hnRNPA2B1 expression through the activation of NF‐κB in GC cells.

### 2.3 hnRNPA2B1 Remodels Metabolic Reprogramming in GC Under *H. pylori* Infection

**Figure 3:**
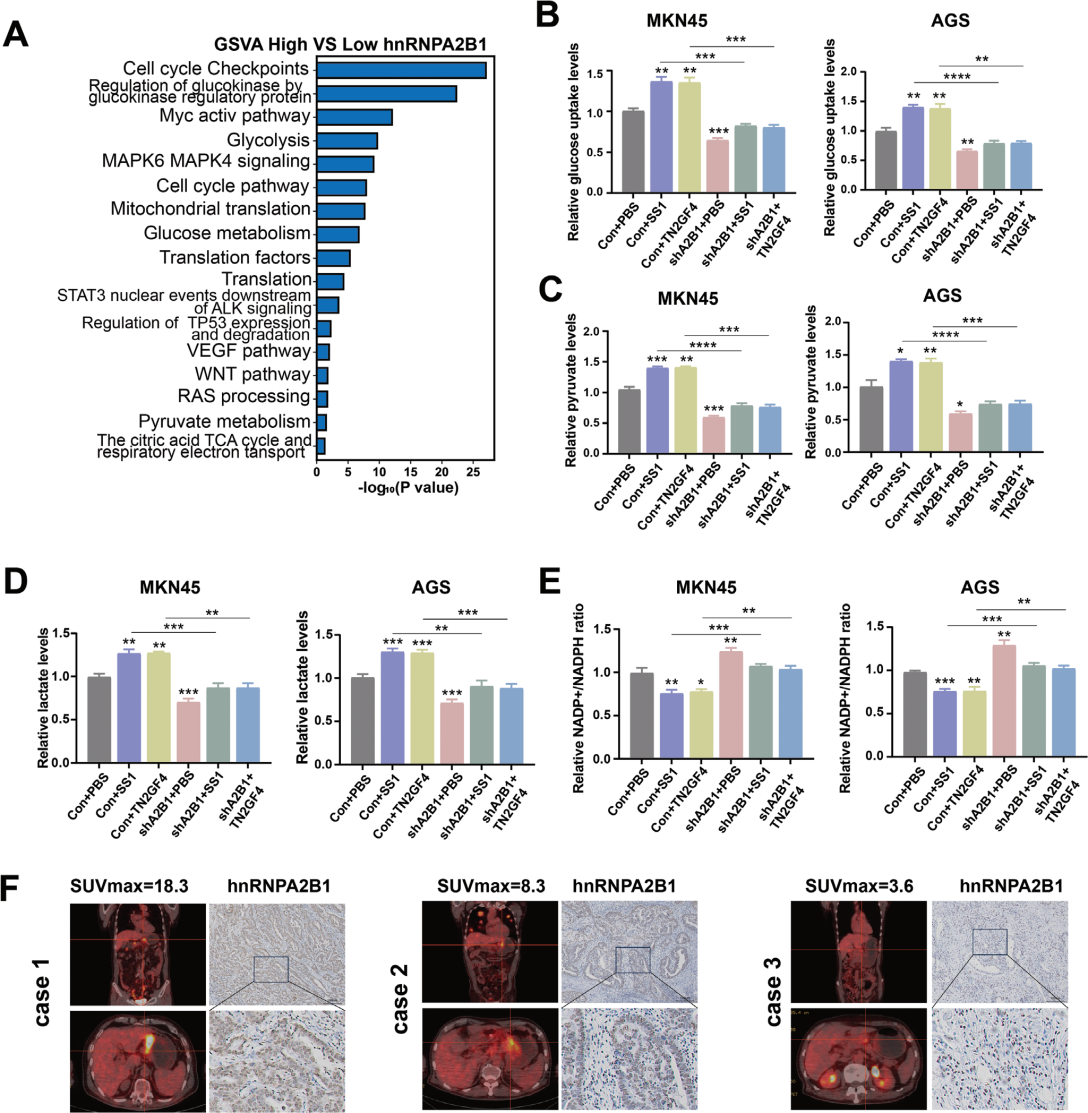
hnRNPA2B1 remodeled metabolic reprogramming in GC cells under *H. pylori* infection. A) GSVA analysis of genes divided into hnRNPA2B1‐high group versus hnRNPA2B1‐low group in the TCGA. (B‐E) The effects of hnRNPA2B1 KD on glucose uptake B), pyruvate C) and lactate production(D), and NADP+/NADPH ratio E) in MKN45 and AGS cells uninfected or infected with *H. pylori* (SS1 and TN2GF4) were determined, respectively. F) Representative ^18^F‐FDG PET/CT images in GC patients exhibiting varying expression of hnRNPA2B1. ^*^ *p* < .05, ^**^ *p* < .01, ^***^ *p* < .001, ^****^ *p* < .0001.

To understand how hnRNPA2B1 contributes to gastric tumorigenesis, we performed Gene Set Variation Analysis (GSVA) using TCGA, comparing the cohorts with high versus low hnRNPA2B1 expression to identify variations in biological functions. It was revealed that hnRNPA2B1 was involved in cell cycle, metabolic reprogramming, and numerous signaling pathways associated with gastric tumorigenesis (**Figure** **3**A). Considering *H. pylori* inducing hnRNPA2B1 expression in GC, we investigated whether *H. pylori* infection could mediate hnRNPA2B1 to modulate tumor metabolism by assessing the alterations in glucose uptake, the production of pyruvate and lactate, and NADP+/NADPH ratios. The results unveiled that *H. pylori*‐induced increase in tumor glucose uptake and lactate and pyruvate production was abolished by hnRNPA2B1 knockdown (KD) in GC cells (Figure 3B–D). Additionally, hnRNPA2B1 KD reversed the *H. pylori*‐induced reduction in NADP+/NADPH ratios in GC cells (Figure 3E). Furthermore, the overexpression of hnRNPA2B1 increased tumor glucose uptake and lactate and pyruvate production in GC cells (Figure S1A–C, Supporting Information), but decreased the NADP+/NADPH ratios (Figure S1D, Supporting Information). To assess the clinical significance of hnRNPA2B1 in patients with GC, we investigated the hnRNPA2B1 expression levels across different SUVmax values, an indicator of tumor glycolytic metabolism measured via PET/CT (18F‐FDG, 18F‐fluorodeoxyglucose) scans in GC. The results indicated an association between hnRNPA2B1 expression and SUVmax value in patients with GC (Figure 3F). These data suggested that hnRNPA2B1 could modulate metabolic reprogramming in response to *H. pylori* infection in GC.

### 2.4 hnRNPA2B1 KD Restricts GC Metastasis and Promotes CDDP Chemosensitivity

**Figure 4:**
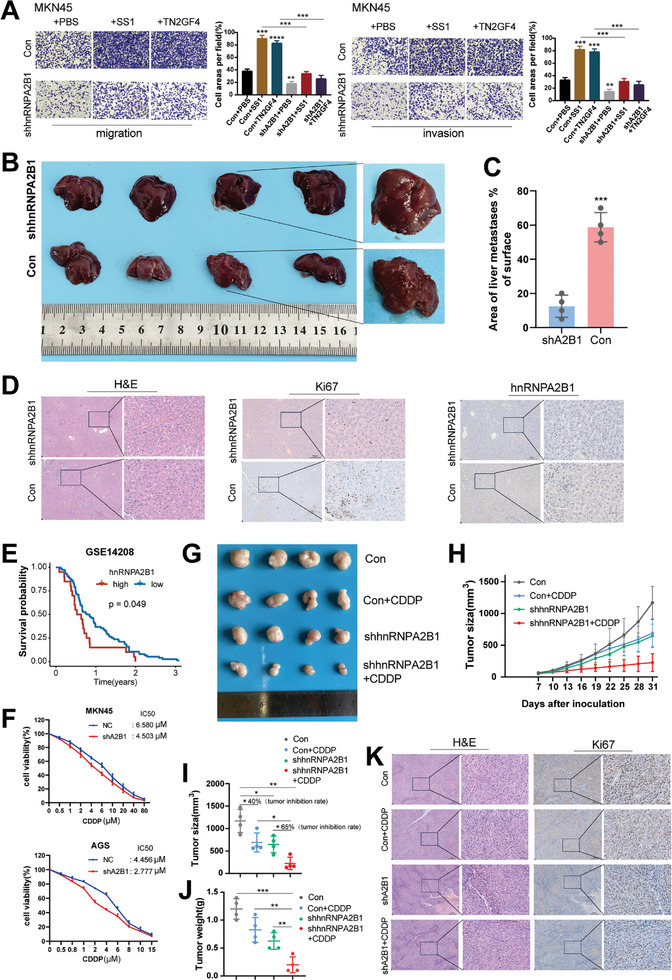
hnRNPA2B1 KD reduced GC invasion and metastasis and favored CDDP chemosensitivity. A) Transwell analysis of the effects of hnRNPA2B1 KD on cell migration (left) and invasion (right) abilities in uninfected or *H. pylori* (SS1 and TN2GF4) infected MKN45 cells. B) Images (left) and representative images (right) of metastatic liver tumors from mice receiving spleen injection with MKN45‐Con and MKN45‐shhnRNPA2B1 cells (n = 4 per group). C) The percentages of liver surfaces occupied by macro‐metastatic nodules were quantified (n = 4 per group). D) Representative images of H&E staining and Ki67, hnRNPA2B1 IHC staining for metastatic liver tumors. E) A negative correlation of hnRNPA2B1 high expression with the OS of GC patients undergoing cisplatin‐based chemotherapy in GEO datasets. (F) Effects of hnRNPA2B1 KD on the viability of MKN45 and AGS cells treated with CDDP were detected by CCK8 assays. (G) The effects of shhnRNPA2B1 combined with CDDP treatment on tumor growth of MKN45 cells (n = 4 per group). H–J) Tumor growth curve H) Tumor volume I) and weight J) were measured. K) Representative images of H&E staining and Ki‐67 IHC staining for xenograft tumors. ^*^ *p* < .05, ^**^ *p* < .01, ^***^ *p* < .001.

According to GSVA analysis, hnRNPA2B1 is involved in numerous signaling pathways associated with tumorigenesis and metastasis. Given that hnRNPA2B1 expression could be induced by H. *pylori* infection, we sought to determine whether *H. pylori* could mediate hnRNPA2B1 to enhance GC metastasis. Transwell analysis revealed that the hnRNPA2B1 KD markedly attenuated *H. pylori*‐induced enhancement in migration and invasion of MKN45 and AGS cells (**Figure** **4**A; Figure S2, Supporting Information). Moreover, we established a murine liver metastasis model by injecting MKN45 cells into the spleens of BALB/C‐nude mice. Notably, the results indicated that the area of liver metastasis was reduced upon hnRNPA2B1 KD (Figure 4B,C; Figure S3A, Supporting Information). Representative liver sections stained with H&E and IHC staining for Ki67 and hnRNPA2B1 were presented in Figure 4D.

In the light of the role of hnRNPA2B1 in modulating tumor metabolic reprogramming, we sought to investigate whether hnRNPA2B1 acts in modulating chemosensitivity in GC. To address this, we used GSE14208 consisting of GC patients treated with cisplatin‐based chemotherapy. As shown in Figure 4E, patients with high hnRNPA2B1 expression had a significantly worse response to chemotherapy than those with low hnRNPA2B1 expression (*p* <0.05). To substantiate this intriguing clinical observation in a laboratory setting, we performed cytotoxicity assays, which indicated that hnRNPA2B1 KD substantially enhanced the sensitivity of GC cells to cisplatin (CDDP) treatment, as reflected by a notable decrease in the half maximal inhibitory concentration (IC_50_) values (Figure 4F). Additionally, overexpression of hnRNPA2B1 resulted in increased half maximal inhibitory concentration (IC50) values, indicating that hnRNPA2B1 overexpression significantly reduced the sensitivity of GC cells to cisplatin (CDDP) treatment (Figure S4, Supporting Information). Following these, we established a tumor xenograft model in vivo employing shhnRNPA2B1 and Con to corroborate the cytotoxicity observations. This model was integrated with CDDP therapy to evaluate the impact of hnRNPA2B1 inhibition on chemotherapeutic efficacy. After CDDP treatment, we found that shhnRNPA2B1 group achieved a 65% tumor growth inhibition compared to the 40% growth inhibition observed in the control group (Figure 4G‐I; Figure S3B, Supporting Information). The corresponding alterations in tumor weight were observed across different groups (Figure 4J). IHC staining revealed that hnRNPA2B KD promoted CDDP‐caused downregulation of Ki67 in gastric tumors compared to the Con group (Figure 4K). These results highlighted hnRNPA2B1 as a potential target for augmenting chemotherapeutic efficacy.

### 2.5 hnRNPA2B1 Acts as an Oncogene to Enhance mRNA Translation Independent of m^6^A Modification

**Figure 5:**
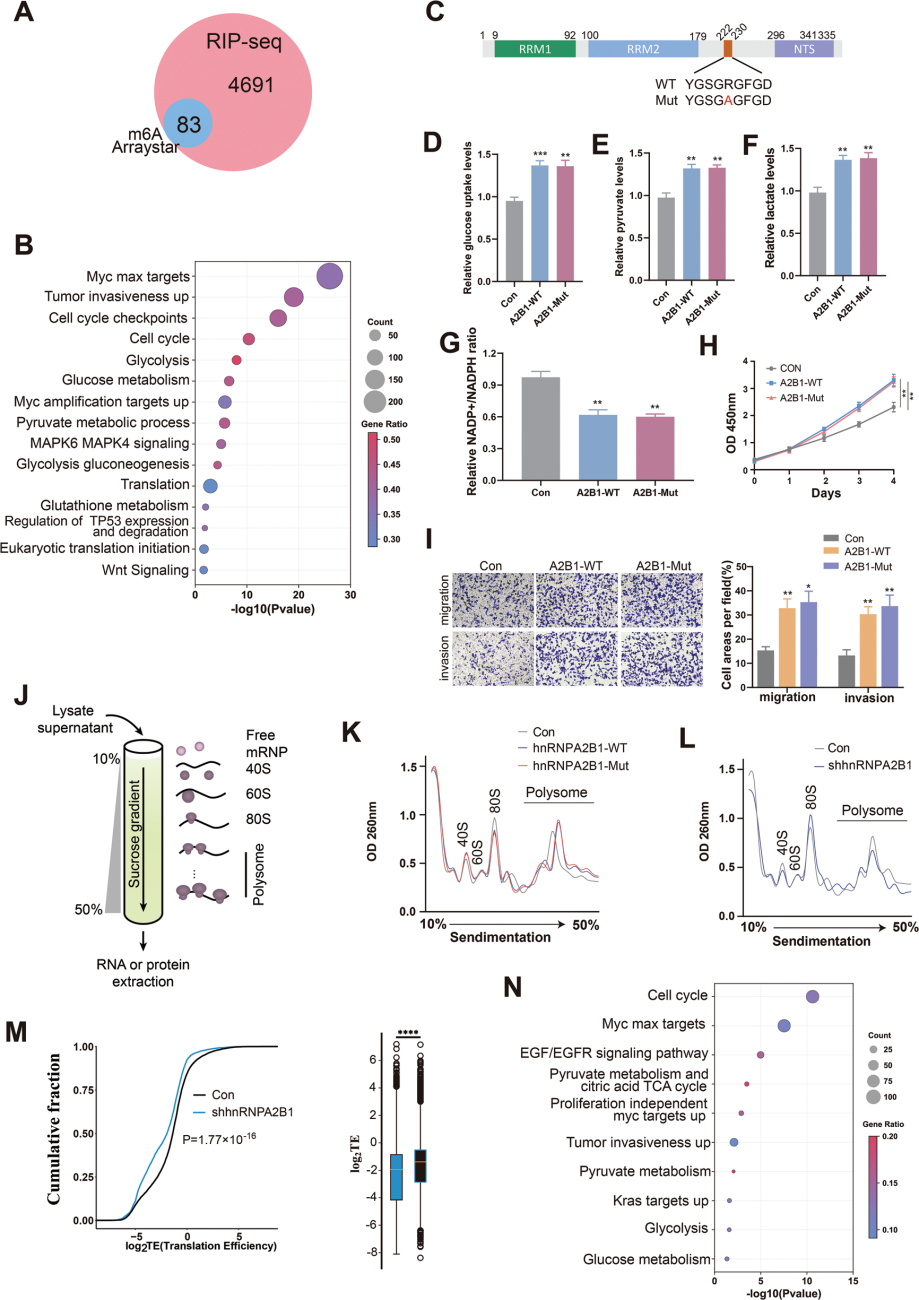
hnRNPA2B1 functioned as an oncogenic regulator of mRNA translation independent of m^6^A modification. A) Comparison between m^6^A‐modified genes regulated by hnRNPA2B1 and hnRNPA2B1‐bound genes identified by hnRNPA2B1 RIP–seq. B) GO analysis of the enriched signaling pathways in “hnRNPA2B1‐only” group (deleting the m^6^A modified genes from hnRNPA2B1‐RIP–seq). C) Schematic diagram of the hnRNPA2B1‐WT and m^6^A catalytically inactive (hnRNPA2B1‐Mut) constructs. (D‐I) The effects of hnRNPA2B1‐WT and hnRNPA2B1‐Mut transduced on glucose uptake D), pyruvate E) and lactate production F), NADP+/NADPH ratio (G), cellular proliferation rates (H), migration and invasion I) in MKN45 cells. J) Schematic of polysome profiling. K) Representative polysome profiling analyses in MKN45 cells transfected with Con, hnRNPA2B1‐WT, and hnRNPA2B1‐Mut. L) Representative polysome profiling analyses in MKN45 cells transfected with Con and shhnRNPA2B1. M) Cumulative‐distribution‐function plot depicting log_2_fold changes of translation efficiency between shhnRNPA2B1 and control groups. N) GO analysis of the enriched signaling pathways in hnRNPA2B1 translationally activated genes. ^**^ *p* < .01, ^***^ *p* < .001, ^****^ *p* < .0001.

Considering hnRNPA2B1 as an RNA‐binding protein (RBP) and its capacity as an m^6^A reader, our focus shifted toward the mechanism through which hnRNPA2B1 contributes to GC. Intriguingly, with the overlaps between m^6^A epitranscriptomic microarray and hnRNPA2B1‐RIP‐seq in MKN45 cells, we found only 83 genes modified by hnRNPA2B1 had overlapping m^6^A signals (defined as “Both”) (**Figure** **5**A), accounting for less than 2% of all hnRNPA2B1‐bound transcripts. This limited overlap underscored the selectivity of hnRNPA2B1 in associating with m^6^A‐modified transcripts within GC cells, and this noteworthy observation indicated that hnRNPA2B1 was more likely to modulate mRNAs that do not contain m^6^A modifications in GC cells. Importantly, by GO analysis, we found that the mRNAs in “hnRNPA2B1‐only” group (deleting the m6A modified genes from hnRNPA2B1‐RIP–seq) were enriched in cancer‐related pathways, such as tumor metabolism and translation processes (Figure 5B). This result suggested that hnRNPA2B1 might exhibit m^6^A‐independent oncogenic functions.

To investigate whether the m^6^A activity of hnRNPA2B1 affects GC progression, we introduced a pivotal point mutation (R226A) into hnRNPA2B1^[^ ^23^ ^]^ aiming to eliminate its m^6^A catalytic functionality (Figure 5C). We found that hnRNPA2B1 overexpression increased glucose uptake as well as lactate or pyruvate synthesis but reduced NADP+/NADPH ratio, but the abrogation of m^6^A binding in hnRNPA2B1 did not attenuate the effect of hnRNPA2B1 overexpression on glycolysis reprograming (Figure 5D–G; Figure S5A–D, Supporting Information). Moreover, this m^6^A catalytic site mutation did not impede hnRNPA2B1 overexpression to promote cellular proliferation, migration, and invasion (Figure 5H,I; Figure S5E,F, Supporting Information). These findings suggested that hnRNPA2B1 acted an oncogenic role through an m^6^A‐independent mechanism. hnRNPA2B1 could bind to numerous protein‐coding mRNAs in a non‐m^6^A manner and GO analysis indicated its involvement in translation regulation (Figure 5B). Furthermore, polysome profiling (Figure 5J) verified that hnRNPA2B1 induced an upregulation in translation activity, independent of m^6^A modification (Figure 5K). This result was evidenced by an increase in the pool of polysomes upon WT or mutant m^6^A binding sites in hnRNPA2B1 (Figure 5K), but a reduction in the polysome pool upon hnRNPA2B1 KD in GC cells (Figure 5L). Ribo‐seq and RNA‐seq demonstrated that hnRNPA2B1 KD pronounced a decrease in translation efficiency in GC cells compared with the control group (Figure 5M). GO analysis indicated that hnRNPA2B1 preferentially regulated the translation‐related genes implicated in metabolic processes and tumor invasion (Figure 5N). Collectively, our observations delineated the role of hnRNPA2B1 in the regulation of mRNA translation devoid of m^6^A modifications.

### 2.6 hnRNPA2B1 Interacts with PABPC1‐eIF4F Complex to Promote non‐m^6^A Translation in GC cells

**Figure 6:**
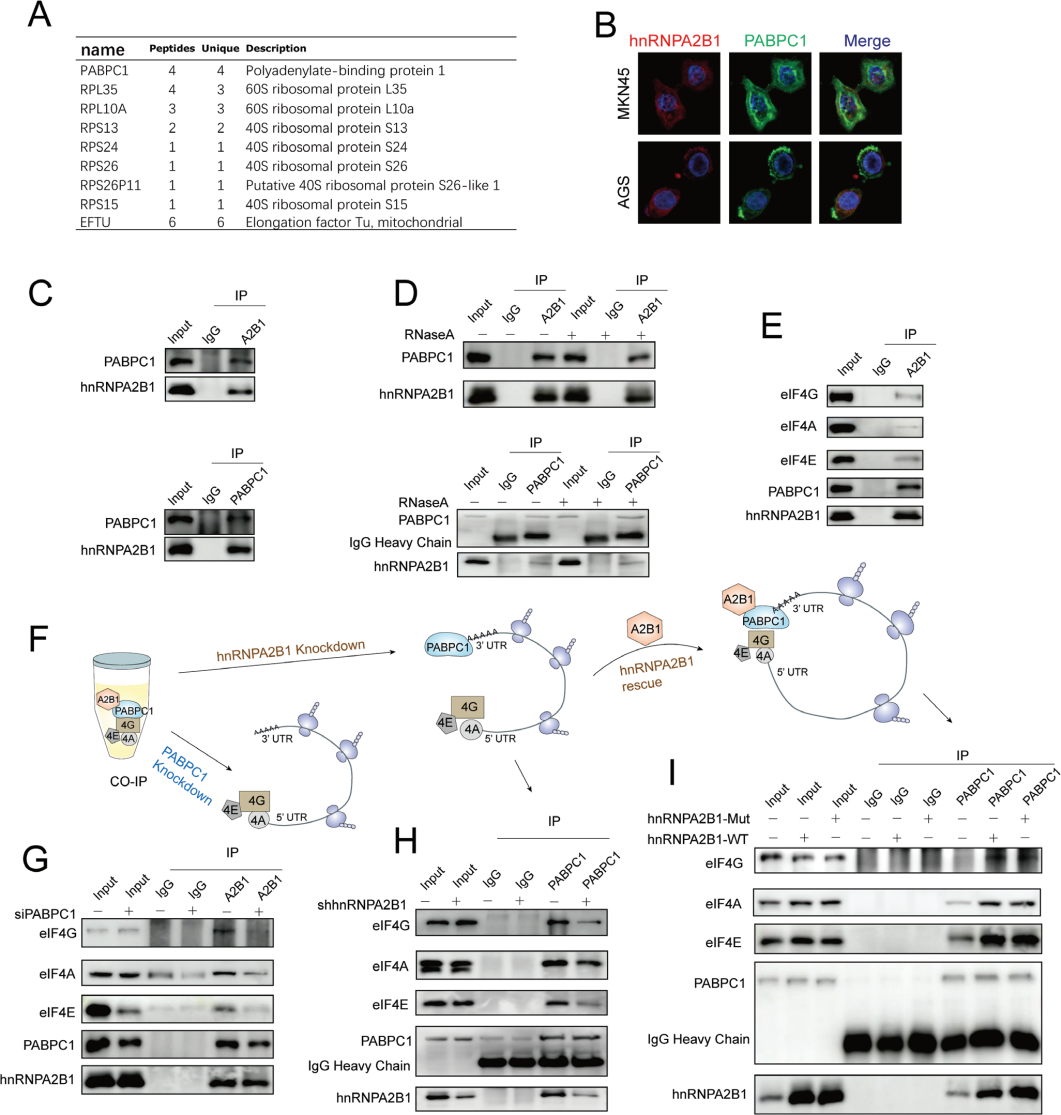
hnRNPA2B1 interacted with PABPC1 to promote the mRNA circularization and non‐m^6^A translation initiation in GC cells. A) List of translation‐related proteins in hnRNPA2B1 interactome identified by MS. B) Immunofluorescence of hnRNPA2B1 (red) colocalized with PABPC1 (green) in MKN45 and AGS. Nuclei were stained with DAPI (blue). C) hnRNPA2B1 Co‐IP with PABPC1 (up) and PABPC1 Co‐IP with hnRNPA2B1 (down) in MKN45 cells. D) hnRNPA2B1 Co‐IP with PABPC1 (up) and PABPC1 Co‐IP with hnRNPA2B1 (down) in MKN45 cells with or without RNase A treatment. E) hnRNPA2B1 Co‐IP with PABPC1 and eIF4F complex. F) Schematic diagram of hnRNPA2B1‐induced translational activation of mRNAs coupled with PABPC1. G) hnRNPA2B1 Co‐IP with PABPC1 and eIF4F complex in MKN45 cells, either with or without si‐PABPC1 treatment. H) PABPC1 Co‐IP with hnRNPA2B1 and eIF4F complex in MKN45‐NC and MKN45‐shhnRNPA2B1 cells. I) PABPC1 Co‐IP with hnRNPA2B1 and eIF4F complex in hnRNPA2B1‐WT or hnRNPA2B1‐Mut transduced hnRNPA2B1 knockdown MKN45 cells.

We have proved the role of hnRNPA2B1 in facilitating mRNA translation. To gain deeper insights into the mechanism underlying hnRNPA2B1‐induced mRNA translation, we took advantage of co‐IP coupled with mass spectrometry (MS) to seek the hnRNPA2B1 interactomes in MKN45 cells. Among the identified hnRNPA2B1‐bound proteins, 9 were categorized as translation‐related proteins, including cytoplasmic poly(A)‐binding protein 1 (PABPC1), 7 ribosomal proteins and eukaryotic translation elongation factor (**Figure** **6**A). GO analysis from RIP‐seq showed that hnRNPA2B1 was implicated in translation initiation, a rate‐limiting step crucial for accurate protein synthesis (Figure 5B). Thus, we hypothesized that hnRNPA2B1 collaborated with PABPC1 to regulate translation initiation in GC cells.

Immunofluorescence analyses in MKN45 and AGS cells demonstrated the subcellular colocalization of hnRNPA2B1 (red) with PABPC1 (green) (Figure 6B). Subsequent Co‐IP assays confirmed the direct interaction between hnRNPA2B1 and PABPC1 in GC cells (Figure 6C; Figure S6A, Supporting Information). Notably, the integrity of their interactions was preserved even in the presence of ribonuclease A (RNase A), indicating its RNA‐independent interaction (Figure 6D; Figure S6B, Supporting Information). PABPC1 plays a critical role in the initiation phase of translation, a key regulatory step in protein synthesis. It binds to the 3′ poly(A) tail of eukaryotic mRNAs and interacts with the 5′ cap‐binding complex eIF4F, consisting of eIF4G, eIF4E, and eIF4A, promoting the formation of a closed‐loop configuration in mRNA^[^ ^21^ ^]^ Continuing this investigation, we explored the association of hnRNPA2B1 with the eIF4F complex, aiming to uncover a novel layer of regulatory interaction in GC. The putative interplay between hnRNPA2B1 and the eIF4F complex was confirmed by Co‐IP, as depicted in Figure 6E and Figure S6C (Supporting Information), substantiating the role of hnRNPA2B1 in the translation process. To unravel the precise function of hnRNPA2B1 within the cap‐eIF4F‐PABPC1‐poly(A) complex (Figure 6F), we knocked down endogenous PABPC1, leading to inhibition of the interaction between hnRNPA2B1 and eIF4F complex (Figure 6G; Figure S6D, Supporting Information). Furthermore, the suppression of hnRNPA2B1 caused a weakened interaction between PABPC1 and eIF4F complex, indicating a relatively relaxed RNA looping configuration in the absence of hnRNPA2B1 (Figure 6H; Figure S6E, Supporting Information). Importantly, the reintroduction of either WT or m^6^A catalytically inactive hnRNPA2B1 effectively promoted the binding between PABPC1 and eIF4F complex (Figure 6I; Figure S6F, Supporting Information). This compelling experimental finding underscored the essential contribution of WT and m^6^A catalytically inactive hnRNPA2B1 to restoring the crucial association between PABPC1 and eIF4F complex in GC cells.

### 2.7 hnRNPA2B1‐PABPC1 Complex Promotes Non‐m^6^A Translation of Epigenetic Factors in GC

**Figure 7:**
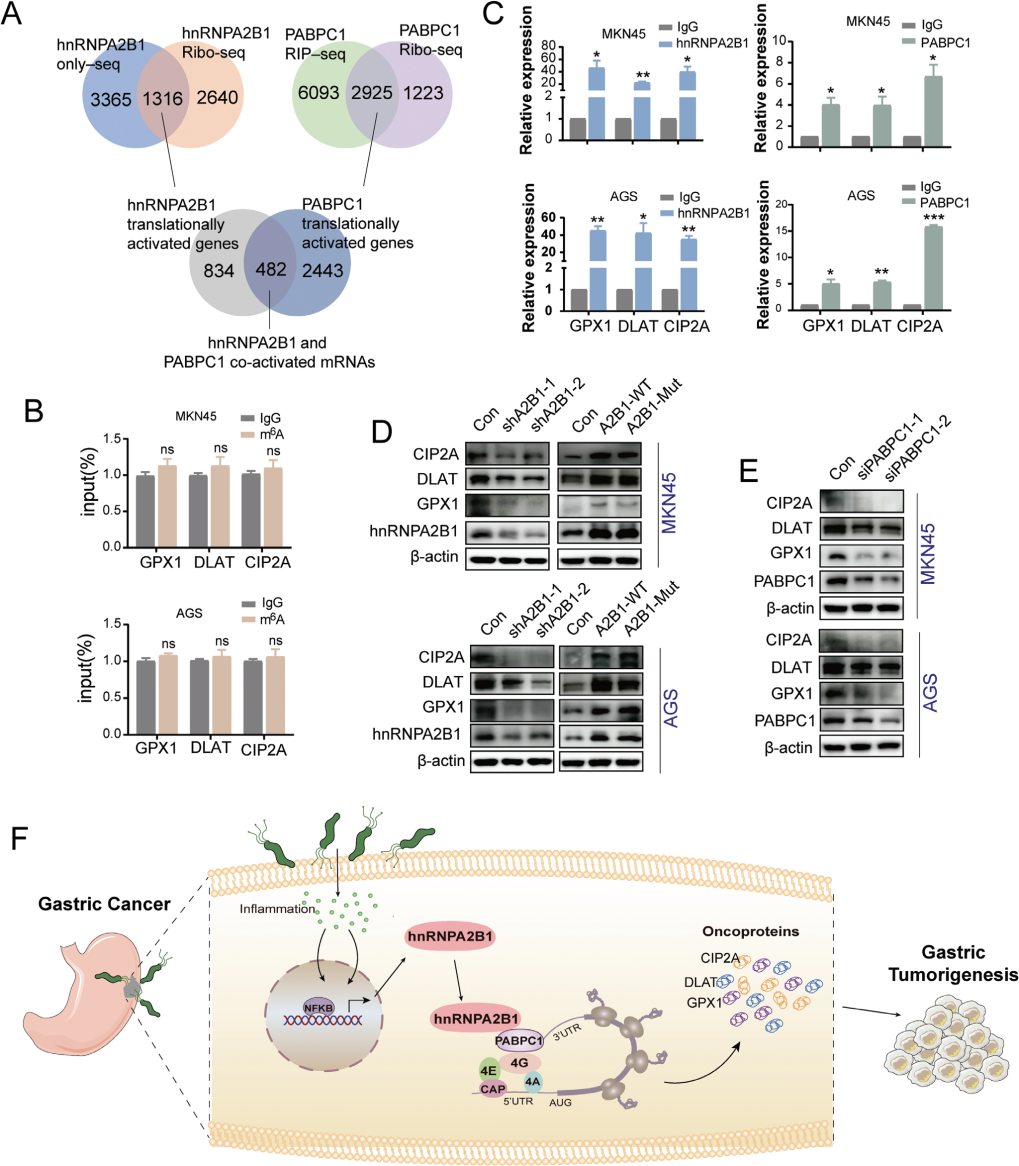
hnRNPA2B1 interacted with PABPC1 to promote the non‐m^6^A translation of oncogenic mRNAs in GC cells. A) Venn diagram depicting the overlap of translationally regulated genes mediated by hnRNPA2B1 through “hnRNPA2B1‐only” and Ribo‐seq and those mediated by PABPC1 by PABPC1‐RIP–seq and Ribo‐seq. B) MeRIP–qPCR analysis of the m^6^A levels of GPX1, DLAT, and CIP2A in MKN45 and AGS cells. C) RIP–qPCR analysis of the enrichment levels of GPX1, DLAT, and CIP2A by hnRNPA2B1 and PABPC1 proteins in MKN45 and AGS cells. D,E) Western blot analysis of GPX1, DLAT, and CIP2A expression in shhnRNPA2B1 (D, left), or hnRNPA2B1‐WT/ hnRNPA2B1‐Mut (D, right) or siPABPC1 E) transfected MKN45 and AGS cells. F) Schematic diagram of the *H. pylori*‐enhanced hnRNPA2B1 promoting the oncogenic mRNA translation by coordinating with PABPC1‐eIF4F complex independent of m^6^A modification in GC progression. ^*^ *p* < .05, ^**^ *p* < .01, ^***^ *p* < .001. ns, not significant.

In the light of the collaborative role of hnRNPA2B1 and PABPC1 in translational modulation, along with the experimentally verified enhancement of GC tumorigenesis by PABPC1,^[^ ^22^ ^]^ we employed an integrated approach to isolate the transcripts under the translational influence of hnRNPA2B1 and PABPC1 by multiple omics “hnRNPA2B1 only”‐seq, Ribo‐seq, and RNA‐seq to pinpoint hnRNPA2B1‐bound mRNAs undergoing translational activation. Simultaneously, we incorporated RIP‐seq and Ribosome‐seq to identify PABPC1‐bound mRNAs experiencing translational activation. The overlapping datasets ultimately revealed 482 transcripts that are commonly activated in translation (**Figure** **7**A). Subsequently, we conducted the selection of well‐established mRNAs, namely CIP2A, DLAT, and GPX1, which were not modified by m^6^A from the overlapping datasets. MeRIP‐qPCR validated an absence of m^6^A signal accumulation on CIP2A, DLAT, and GPX1 in GC cells (Figure 7B), however, RIP assay confirmed that these 3 transcripts unequivocally exhibited the binding by the proteins hnRNPA2B1 and PABPC1 (Figure 7C). Western blot analyses verified that the transfection with hnRNPA2B1‐WT or Mut increased the protein levels of CIP2A, DLAT, and GPX1 (Figure 7D), but hnRNPA2B1 KD (Figure 7D) or PABPC1 KD (Figure 7E) reduced their protein levels in GC cells.

## 3 Discussion

GC poses a serious public threat to human health globally, and screening the therapeutic targets for GC is crucial.^[^ ^1^, ^3^ ^]^ Previous and our studies showed that m^6^A RNA modification is involved in pathogenesis and progression of GC.^[^ ^24^, ^25^ ^]^ hnRNPA2B1 as an m^6^A reader promotes tumor development and progression in a m^6^A‐dependent manner.^[^ ^26^, ^27^ ^]^ In this study, we discovered a novel mechanism by which *H. pylori*‐enhanced hnRNPA2B1 coordinated with PABAC1 to promote mRNA translation and GC progression independent of m^6^A modification.

*H. pylori* as a type I carcinogen infects approximately half of the global population and causes severe gastric diseases including GC ^[^ ^28^ ^]^ Upon *H. pylori* infection, the bacterium can colonize the gastric mucosa, secrete virulence factors like CagA, and trigger chronic inflammation in the gastric epithelium, providing a microenvironment for the initiation and progression of cancerous lesions, wherein NF‐κB signaling can be activated by *H. pylori* infection to induce gastric mucosal inflammation and cancer transformation.^[^ ^15^, ^16^ ^]^ In our study, we identified the upregulation of hnRNPA2B1 in human GC samples compared to the gastritis and intestinal metaplasia with *H. pylori* infection and confirmed that elevated expression of hnRNPA2B1 possessed poor OS in patients with GC. We further found that *H. pylori* infection not only activated NF‐κB signaling, but also recruited NF‐κB to the promoter of hnRNPA2B1, leading to the transcriptional upregulation of hnRNPA2B1 in GC cells. Our results showed that NF‐κB‐hnRNPA2B1 axis might be involved in *H. pylori*‐associated GC tumorigenesis.

Cancer cells autonomously alter their metabolic pathway to support the heightened energy and biosynthesis needs essential for their own proliferation and survival^[^ ^29^ ^]^ Metabolic reprogramming, as a hallmark of cancer malignancy, suggests evolving metabolic characteristics and preferences during tumor progression, potentially contributing to drug resistance.^[^ ^30^, ^31^ ^]^ Recognized as a key obstacle in cancer metastasis, metabolic constraints of glycolysis can limit the dissemination of primary cancer cells.^[^ ^29^, ^30^ ^]^ We herein found that targeting hnRNPA2B1 by shRNA weakened the glycolysis, suppressed tumor growth and liver metastasis but enhanced CDDP chemosensitivity in GC cells. Our findings indicated that hnRNPA2B1 as an oncogenic factor might provide a personalized and effective treatment strategy for GC.

Recent studies have expanded our understanding of hnRNPA2B1 in mediating m^6^A modifications to regulate gene expression at transcriptional and epigenetic levels.^[^ ^27^, ^32^ ^]^ Our investigations revealed that hnRNPA2B1 could interact with over 4000 mRNA transcripts, yet the majority of which lacked the m^6^A RNA modifications. Moreover, we discovered that the cytoplasmic hnRNPA2B1 favored a specific interaction between PABPC1 and the eIF4F complex in GC cells. Notably, this regulatory mechanism did not depend on the influence from m^6^A modification. We offered the pioneering evidence that underscored the novel role of hnRNPA2B1 in tuning protein synthesis by augmenting PABPC1‐eIF4F complex interaction in GC cells.

In addition, it is known that dysregulated mRNA translation, specifically the irregular mRNA circularization driven by PABPC1‐eIF4F complex, acts a pivotal role in the intricate chain of events that precipitate cancer onset.^[^ ^19^, ^21^, ^22^ ^]^ Translation serves as a foundational mechanism exploited by cancer cells to sustain their malignant characteristics^[^ ^18^ ^]^ Our results further elucidated the preferential non‐m^6^A translation of specific mRNAs regulated by hnRNPA2B1‐PABPC1 axis. Among these, GPX1, DLAT and CIP2A stood out due to their roles in carcinogenesis. GPX1, involved in detoxifying reactive oxygen species, allows tumor cells to cope with the oxidative stress that otherwise would limit their survival.^[^ ^33^, ^34^ ^]^ DLAT, as a component of the pyruvate dehydrogenase complex, is a pivotal catalyst in the shift toward aerobic glycolysis – a hallmark of cancer metabolism known as the Warburg effect.^[^ ^35^, ^36^ ^]^ CIP2A acts as a malignancy‐promoting factor through inhibiting protein phosphatase 2A, thereby sustaining the hyperactive state of oncogenic signaling required for continued tumor growth and progression.^[^ ^37^, ^38^ ^]^ Our findings suggest that hnRNPA2B1‐PABPC1 axis might promote the non‐m^6^A translation of GPX1, DLAT and CIP2A in GC cells.

In summary, this study underscores a critical role of hnRNPA2B1 in response to *H. pylori* infection during gastric tumorigenesis, more importantly, we uncover a novel mechanism of hnRNPA2B1 in promoting mRNA translation independent of m^6^A modification. Increased expression of hnRNPA2B1 can decrease chemosensitivity and predict poor prognosis in patients with GC. This research enriches the academic discourse surrounding *H. pylori*‐associated GC and offers novel insights into the potential therapeutic innovations in the realm of GC.

## 4 Experimental Section

### Public Database Analysis

To comprehensively execute gene expression and prognosis analysis, the TCGA‐GC cohort is utlized from the National Cancer Institute (https://portal.gdc.cancer.gov/). A series of human datasets (GSE63089, GSE33335, GSE5081, GSE60662, GSE13911, GSE19826, GSE27342, GSE51575, GSE79973, GSE81948, GSE13195, GSE29272, GSE65081, and GSE122401) were sourced for screening hnRNPA2B1 expression in *H. pylori*‐associated GC from the Gene Expression Omnibus (GEO) DataSets (https://www.ncbi.nlm.nih.gov/gds/). hnRNPA2B1 expression data and Kaplan–Meier survival plots for GC patients treated with cisplatin‐based chemotherapy were obtained from GSE14208. All data were analyzed using R software (R 4.3.1; https://www.r‐project.org/).

### Human GC Tissue Samples

A human tissue microarray (TMA) (STC1602, Qutdo Biotech, Shanghai, China) containing 82 cases of GC samples was used for validation of the expression and prognosis of hnRNPA2B1 in GC. 20 pairs of *H. pylori*‐positive or negative GC samples were confirmed by a combination of pathologic diagnosis and ^13^C urea breath test. This study protocol was approved by the Ethics Committee of Shanghai Sixth People's Hospital.

### Immunohistochemical (IHC) Analysis

IHC staining was performed to validate the protein levels of hnRNPA2B1 in GC. Briefly, paraffin‐embedded tissue sections were first deparaffinized, followed by antigen retrieval using citric acid buffer. Blocking was performed using 3% hydrogen peroxide and 5% bovine serum albumin (BSA). After overnight incubation with the anti‐hnRNPA2B1 antibody (shown in Table S1, Supporting Information) at 4 °C, the slides were incubated for 20 min at room temperature with horseradish peroxidase (HRP)‐labeled polymer conjugated to a secondary antibody (Max Vision Kit), followed by a brief 2 min diaminobenzidine (DAB) treatment. Finally, the nuclei were counterstained with hematoxylin. The percentage of stained cells was scored as follows: 0% as 0, 1%–10% as 1, 10%–30% as 2, 31%–50% as 3, 51%–75% as 4, and 76–100% as 5, and the intensity of staining: no staining as 0, weak staining as 1, moderate staining as 2, strong staining as 3.

### Cell Culture, Cell Transfection, and Lentivirus Infection

Human GC cell lines were purchased from the Cell Bank of the Shanghai Institute for Biological Science (Shanghai, China). Cells were cultured in RPMI‐1640 medium or DMEM medium (Gibco, Thermo Fisher, USA) supplemented with 10% FBS (Gibco) and 1% penicillin/streptomycin (Invitrogen, Carlsbad, CA, USA) at 37 °C in a humidified atmosphere with 5% CO_2_. Then, the transient transfections of PABPC1 siRNA (si‐PABPC1, shown in Table S3, Supporting Information) from RiboBio (Guangzhou, China) or wild type (WT)/mutant (Mut) hnRNPA2B1 plasmids from Hanbio (Shanghai, China) in adherent cells were performed using Lipofectamine 3000 reagent (Invitrogen) according to the manufacturer's instructions. Lentiviral vectors for hnRNPA2B1 shRNA (shA2B1, shown in Table S3, Supporting Information) or empty control (Con) were purchased from Hanbio (Shanghai, China), and the lentivirus‐infected cells were selected with puromycin to generate stable cell lines.

### RNA isolation and real‐time quantitative PCR (RT‐qPCR)

Total RNA was extracted from GC cell lines using TRIzol reagent (Vazyme, China) and then reversely transcribed to complementary DNA using HiScript III 1st Strand cDNA Synthesis Kit (Vazyme, China) and ChamQ Universal SYBR qPCR Master Mix (Vazyme, China). All primers used for amplifying target genes were listed in Table S2 (Supporting Information).

### Cell Proliferation Assay

MKN45 and AGS cells were seeded into 96‐well plates at 2000 cells per well. Cell proliferation was assessed using a 10% CCK‐8 (Vazyme) solution diluted in culture medium without FBS, with an incubation period of 2 h at 37 °C. Measurements of proliferation rates at 0, 24, 48, 72, and 96 h post‐treated were performed on a BioTek microtitre plate reader using the recommended protocol from the manufacturer.

### Cell Migration and Invasion Assays

MKN45 and AGS cells were seeded into transwell inserts with polyester membranes for migration assays (Corning Costar) or Matrigel‐coated chambers for invasion studies (Corning Costar) in non‐FBS medium. Below each chamber, 600 µl of 10% FBS‐containing medium was added. Following incubation for 20 h, cells traversing the membranes were crystal violet‐stained and enumerated at 20× magnification.

### *H. Pylori* Strains

*H. pylori* strains SS1 and TG2NF4 were used for infecting the GC cell lines. They were cultured on Columbia blood agar plates (Comagal, Shanghai, China) and in Brucella broth (BD Biosciences) supplemented with 10% FEB for 16 to 18 h at 37 °C under an atmosphere of 10% CO_2_. In subsequent in vitro assays, GC cells were incubated with the *H. pylori* strains at a 100:1 multiplicity of infection (MOI).

### Cleavage Under Targets and Tagmentation (CUT&Tag)

CUT&Tag assay was performed following the manufacturer's instructions (Vazyme, TD904). Cells were collected and washed with wash buffer. Subsequently, they were adhered to ConA beads for 10 min at room temperature. On the second day, secondary antibodies were added and incubated with rotation for 60 min at room temperature. Then, the samples were washed three times with DIG wash buffer, followed by the addition of pA/G‐Tnp and incubation at room temperature for 60 min. Then, Dig‐300 Buffer and TTBL were added, followed by incubation at 37 °C for 1 h to fragment the DNA. After fragmentation, DNA extract beads were added to each sample. After incubation at room temperature for 20 min, beads were rinsed with B&W Buffer. Following this, DNA was eluted with ddH2O and qPCR was employed for quantitative analysis.

### Co‐Immunoprecipitation (IP) and Western Blot Analysis

Cells were washed with ice‐cold PBS and mixed with the elution buffer following the manufacturer's instructions (Proteintech, USA). The Co‐IP proteins were heated with loading buffer and separated by SDS‐PAGE following the protocols of the western blot assays.

Western blot analysis was performed according to the standard protocol. Briefly, GC cells were obtained and extracted using RIPA Lysis buffer (Beyotime, Shanghai, China) and separated on SDS‐PAGE gels (EpiZyme, Shanghai, China), and then they were transferred from SDS‐PAGE gels to PVDF membranes (Millipore PVDF 0.45 mm), followed by incubation with appropriate primary and secondary antibodies (shown in Table S1, Supporting Information).

### Measurement of Glucose Uptake, Lactate, and Pyruvate Production and NADP+/NADPH Ratio

Quantifications were measured using Glucose Uptake Assay Kit (DOJINDO, Japan), pyruvate and Lactate Assay Kit (Nanjing Jiancheng Bioengineering Institute), NADP+/NADPH Colorimetric Assay Kit (Elabscienc, China) according to the manufacturers’ protocols, respectively.

### Immunofluorescence

Cells were seeded on glass‐bottom culture cell dish and grown for at least 16 h before fixation. The cells were fixed with 4% paraformaldehyde and permeabilized with 0.1% Triton X‐100. Subsequently, the cells were blocked using 5% bovine serum albumin. They were then stained overnight at 4 °C with the desired primary antibodies, diluted 1:100 in the blocking solution. Following the washing step with PBS, cells underwent a 1 h incubation at room temperature with either goat anti‐rabbit IgG H&L conjugated to Alexa Fluor 594 (shown in Table S1, Supporting Information) or goat anti‐mouse IgG H&L tagged with CoraLite488 (shown in Table S1, Supporting Information). After nuclear staining with DAPI, images were captured using a confocal microscopy.

### Polysome Profiling

Cells were treated with 100 µg mL^−1^ cycloheximide (MCE) for 5 min at 37 °C. Then, cells were washed and scraped with ice‐cold 1x PBS containing 100 µg mL^−1^ cycloheximide. Lysis buffer was then utilized for preparing cell suspensions on ice. Supernatant was collected after a centrifugation process of cell suspensions at 15 000 rpm for 5 min at a temperature of 4 °C. The same OD amount of supernatant was loaded onto 10% to 50% sucrose gradients followed by ultracentrifugation at 35 000 rpm for 2 h at 4 °C using SW40Ti rotor in a Beckman Coulter. Then Gradients were fractionated and monitored at absorbance 260 nm.

### Ribosome Profiling Sequencing (Ribo‐seq)

Ribo‐seq was performed by CloudSeq Biotech (Shanghai, China). Cells were incubated with 100 mg mL^−1^ of cycloheximide for 5 min and then treated then lysed by polysome buffer. The RNA outside of the ribosomes was digested with nucleases, only leaving the RNA fragments that were protected by the ribosomes. Ribosomes with the protected RNA fragments were separated from other cell components, The recovered RNA fragments were converted into a library of cDNA (complementary DNA) through reverse transcription. The cDNA library was sequenced using next‐generation sequencing technology (Illumina NovaSeq 6000 instrument).

### RNA Immunoprecipitation (RIP) Assay and RIP‐Seq

MKN45 cells were seeded in 10‐cm dish at 70%–80% confluency and harvested by trypsinization. For antigen capture, magnetic beads conjugated with either PABPC1, hnRNPA2B1, or IgG antibodies (shown in Table S1, Supporting Information) were utilized, employing the RIP kit (Geneseed Biotech, Guangzhou, China), The coprecipitated RNAs were extracted, and RT‐qPCR was utilized for verification.

Following the RIP assay, pull‐down RNA fragments was collected, rRNAs were removed from the immunoprecipitated RNA samples by using Ribo‐Zero rRNA Removal Kit (Illumina, San Diego, CA, USA). RNA libraries were constructed by using rRNA‐depleted RNAs with TruSeq Stranded Total RNA Library Prep Kit (Illumina, San Diego, CA, USA) according to the manufacturer's instructions. Libraries were controlled for quality and quantified using the BioAnalyzer 2100 system (Agilent Technologies, Inc., USA). Libraries (10pM) were denatured as single stranded DNA molecules, captured on Illumina flow cells, amplified in situ as clusters and finally sequenced for 150 cycles on an Illumina HiSeq Sequencer according to the manufacturer's instructions at Cloudseq (Shanghai, China).

### RNA Sequencing (RNA‑Seq)

High‐throughput RNA‐seq was performed by CloudSeq Biotech (Shanghai, China). Briefly, rRNA was removed from total RNA using a NEBNext rRNA Depletion Kit (New England Biolabs, Inc., Massachusetts, USA) following the manufacturer's instructions. RNA libraries were constructed by using a NEBNext Ultra II Directional RNA Library Prep Kit (New England Biolabs, Inc., Massachusetts, USA) according to the manufacturer's instructions. Libraries were controlled for quality and quantified using a BioAnalyzer 2100 system (Agilent Technologies, Inc., USA). Library sequencing was performed on an Illumina NovaSeq 6000 instrument with 150 bp paired‐end reads. Paired‐end reads were harvested from Illumina NovaSeq 6000 sequencer and were quality‐controlled by Q30. After 3′ adaptor trimming and removal of low‐quality reads with Cutadapt software (v1.9.3), the high‐quality clean reads were aligned to the reference genome (UCSC hg19) with HISAT2 software (v2.0.4). Then, guided by the Ensembl gtf gene annotation file, the Cuffdiff tool (part of Cufflinks) was used to obtain gene expression in FPKM. Fold change (FC) and *P* values were calculated based on the FPKM values to identify the differentially expressed mRNAs.

### m^6^A‐RNA Immunoprecipitation (MeRIP)‐qPCR

Total RNAs from MKN45 cells were isolated via TRIzol (Invitrogen), followed by mRNA purification using the Seq‐StarTM poly(A) Isolation Kit (Arraystar, Rockville, United States). RNA samples were fragmented into 100 nucleotides fragments and then underwent m^6^A IP using ABE572 antibody (Merck Millipore, Germany), according to the standard protocol of the MeRIP m^6^A kit (Merck Millipore, Germany). Afterward, RNA was analyzed by RT‐qPCR.

### Animal Experiments

Female BALB/C‐nude mice (5 to 6 weeks old) were used for in vivo subcutaneous xenograft tumor and liver metastasis models. MKN‐45 cells (6 × 10^6^) transfected with Control or sh‐hnRNPA2B1 lentiviruses were injected subcutaneously into the right flank of nude mice. After 1 week of inoculation, mice were randomly divided into four groups (4 mice/group) and treated as follow: 1) Control with PBS treatment; 2) Control with CDDP treatment; 3) sh‐hnRNPA2B1 with PBS treatment; 4) sh‐hnRNPA2B1 with CDDP treatment. CDDP was delivered 3 mg k^−1^g per 3 days, intraperitoneally. The effect was determined by tumor volume and tumor weight. The volume(mm^3^) = length × width^2^ × 0.5.

For the liver metastasis model, mice were randomly divided into 2 groups (n = 4 per group) and 2 × 10^6^ MKN‐45 cells were injected into the spleen of the BALB/c nude mice. After 2 months, the mice were sacrificed to harvest liver/tumor tissues for analysis. The animal experiments were approved by the Ethics Committee of Shanghai Sixth People's Hospital.

### Enrichment Analysis Related to Pathway and Function

Gene Set Enrichment Analysis (GSEA) and Gene Set Variation Analysis (GSVA) were important tools for enrichment analysis. For GSEA The Normalization Enrichment Score (NES) and the false discovery rate (FDR) of each gene set were calculated using the “cluster Profiler” (v3.12.0) package. GSVA was used to perform GSVA on high‐risk and low‐risk subgroups using the “GSVA” R package. In this study, both methods were used to analyze the pathways and functions associated with hnRNPA2B1. GO functional enrichment analysis was performed by R package clusterProfiler.

### Statistical Analysis

Survival curves were generated using the Kaplan‐Meier method and log‐rank testing. Two groups were compared using Student's *t* test and analysis of variance. Cell biology experimental data were obtained from at least three independent experiments. Most analyses were analyzed using R software 4.1.1 and GraphPad Prism 8.3.0, where *p*‐value < 0.05 was regarded as statistical significance, and the values were shown as mean ±SD.

## Conflict of Interest

The authors declare no conflict of interest.

## Author Contributors

Y.Y. and Y.‐L.Y. contributed equally to this work. J.Z., J.S.Z., and YY conceived the study questions and designed the study. Y.Y. and Y.L.Y. performed the experiments and analyses. X.Y.C. and Z.Y.C. collected samples and laboratory data. Y.Y. drafted the manuscript. J.Z., J.S.Z., and Y.Y. supervised the study. All authors have read and approved the manuscript.

## Supporting information

Supporting Information

## Data Availability

The data that support the findings of this study are available from the corresponding author upon reasonable request.
