# Supplementary material for: Helicobacter Pylori‐Enhanced hnRNPA2B1 Coordinates with PABPC1 to Promote Non‐m6A Translation and Gastric Cancer Progression
Source: Adv Sci (Weinh). 2024 Jun 17;11(30):2309712. doi: 10.1002/advs.202309712 (PMC11321670; doi:10.1002/advs.202309712)
Supplement: Supplementary file 1 — Supporting Information [file ADVS-11-2309712-s001.docx]

**Supplementary Figures and Figure legends**


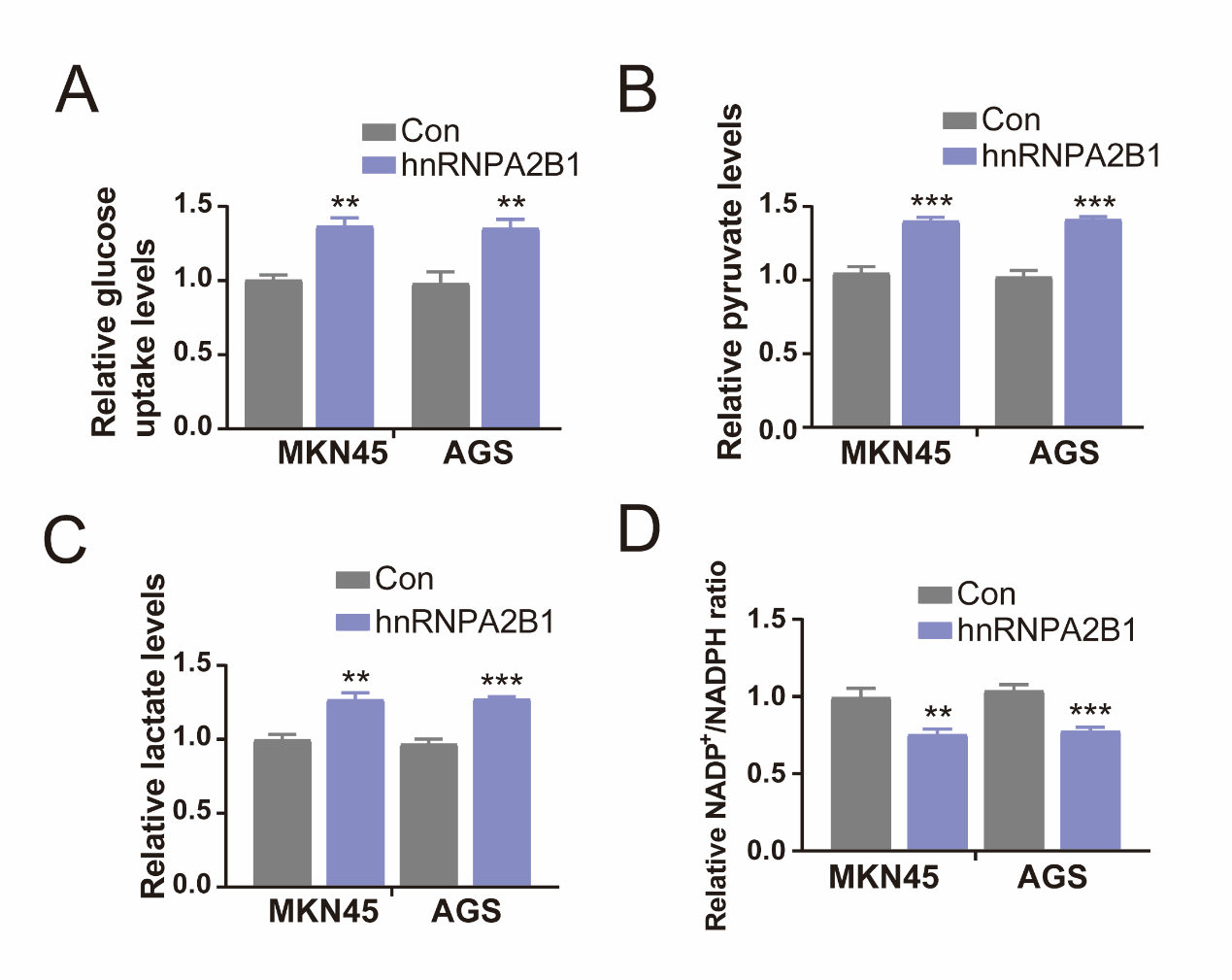


**Figure S1** The effects of hnRNPA2B1 overexpression on glucose uptake (A), pyruvate (B) and lactate production (C), NADP+/NADPH ratio (D) in GC cells


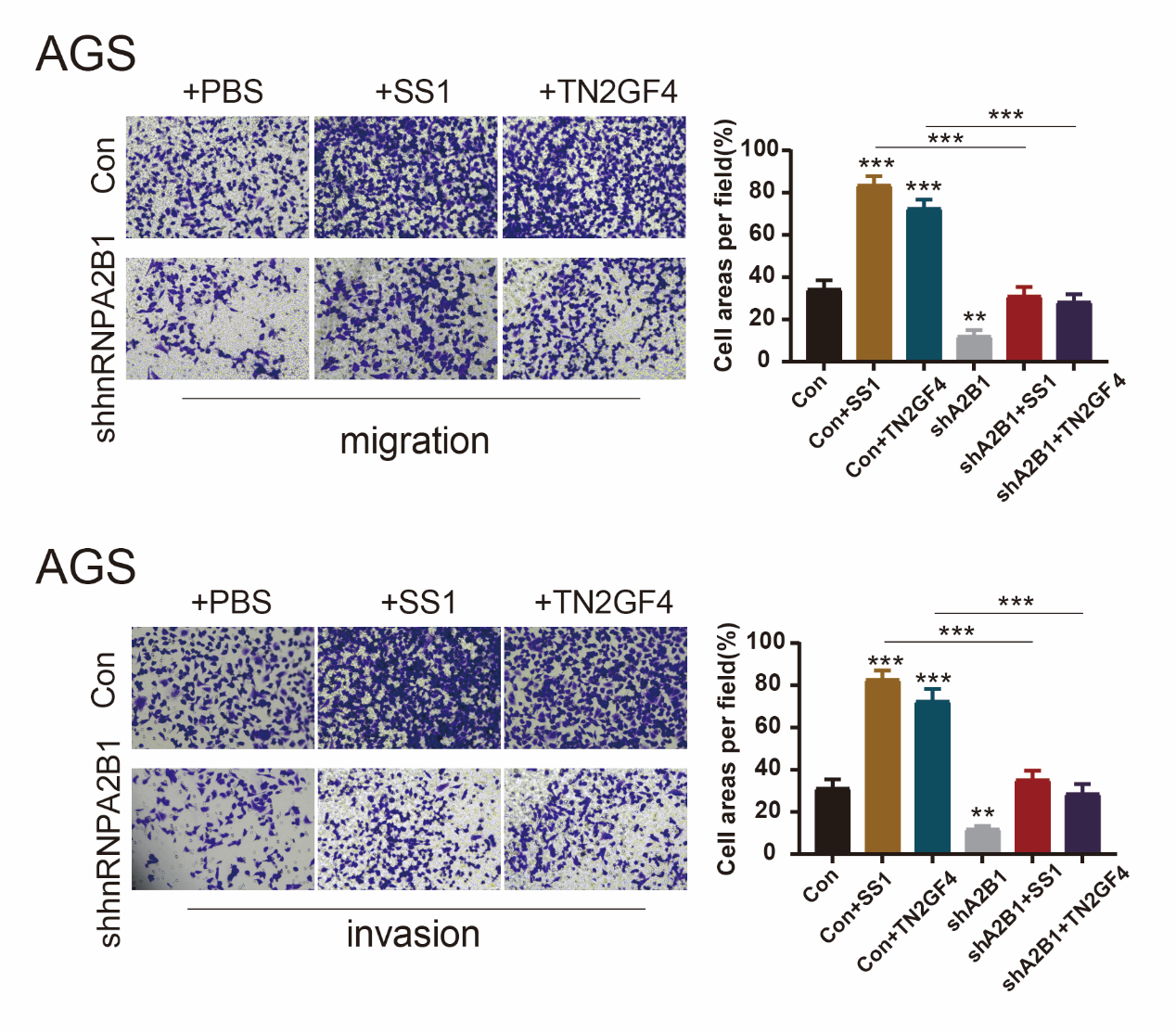
**Figure S2.** Transwell analysis of the effects of hnRNPA2B1KD in AGS cells. Transwell analysis of the effects of hnRNPA2B1 KD on cell migration (up) and invasion (down) abilities in uninfected or *H. pylori* (SS1 and TN2GF4) infected AGS cells.


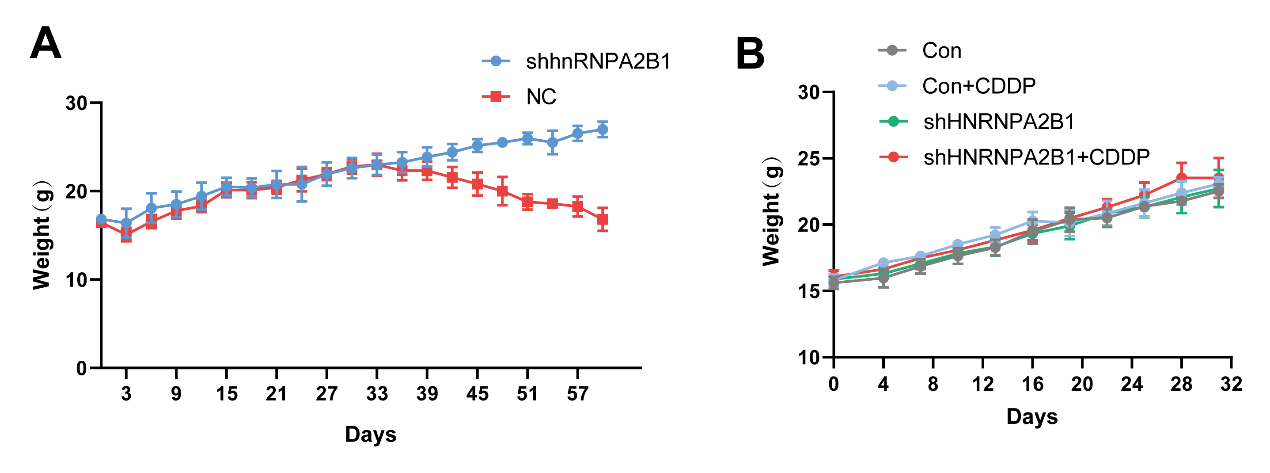


**Figure S3.** Weight changes in mice with liver metastasis model (A) and tumor xenograft model with or without CDDP treatment (B)


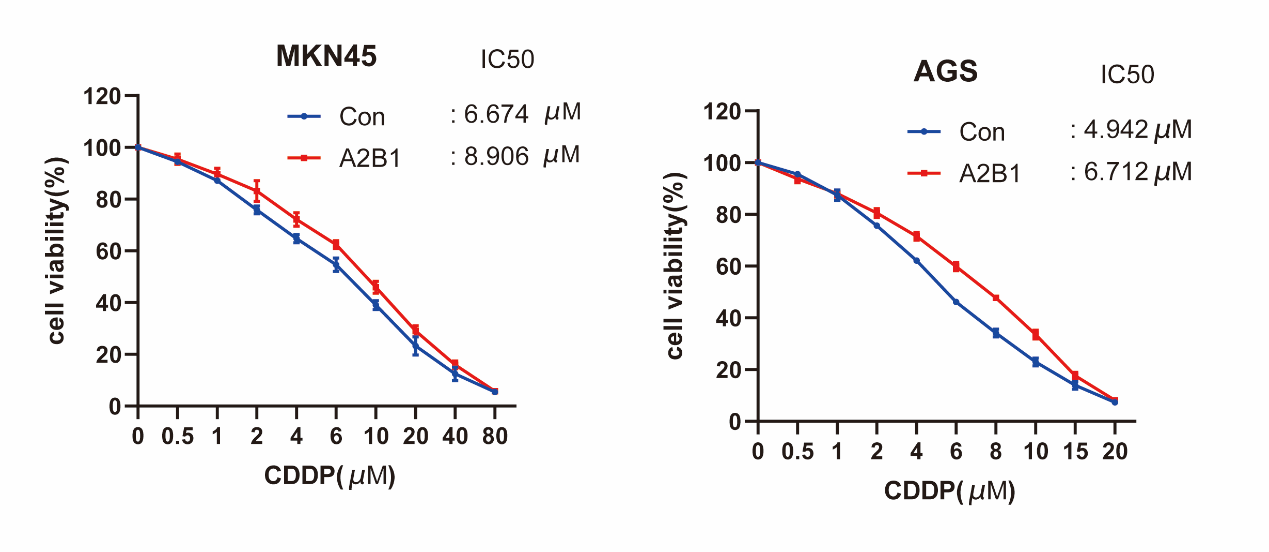


**Figure S4.** Effects of hnRNPA2B1 OE on the viability of MKN45 and AGS cells treated with CDDP were detected by CCK8 assays.


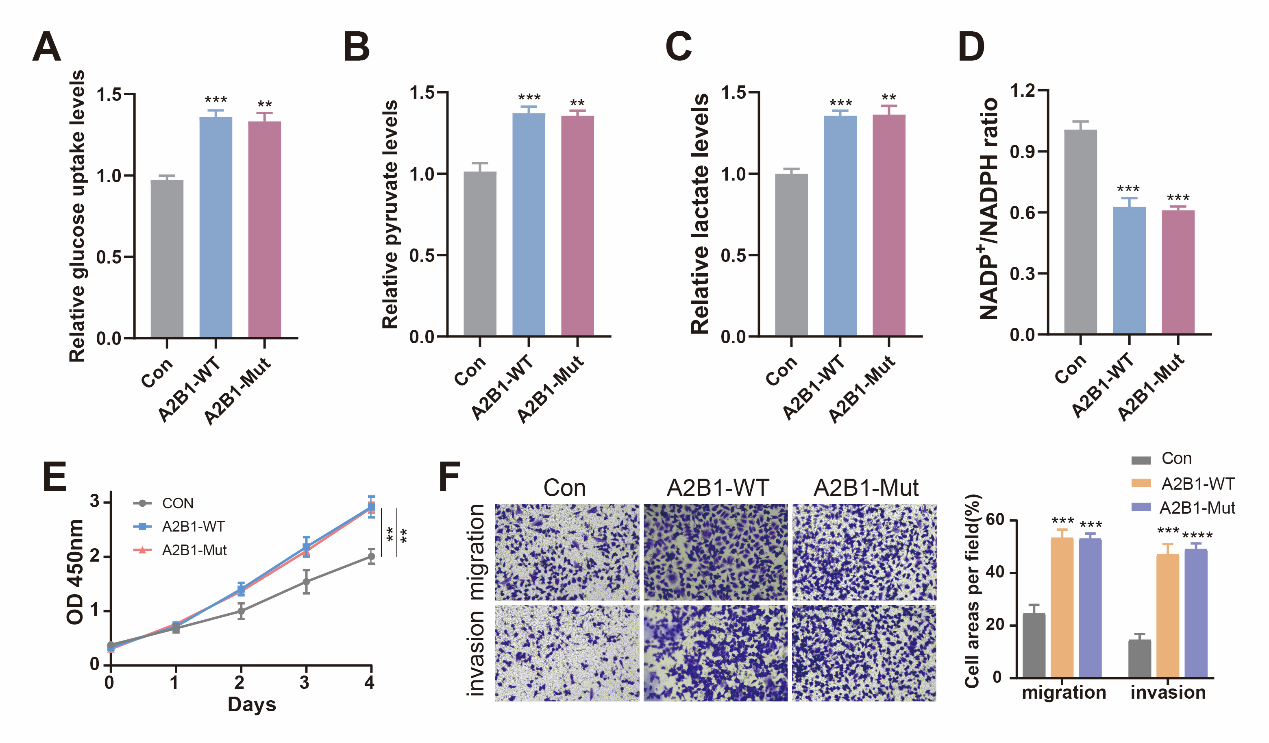
**Figure S5.** The effects of hnRNPA2B1-WT and hnRNPA2B1-Mut in AGS cells. (A-F) The effects of hnRNPA2B1-WT and hnRNPA2B1-Mut transduced on glucose uptake (A), pyruvate (B) and lactate production (C), NADP+/NADPH ratio (D), cellular proliferation rates (E), invasion and migration (F).


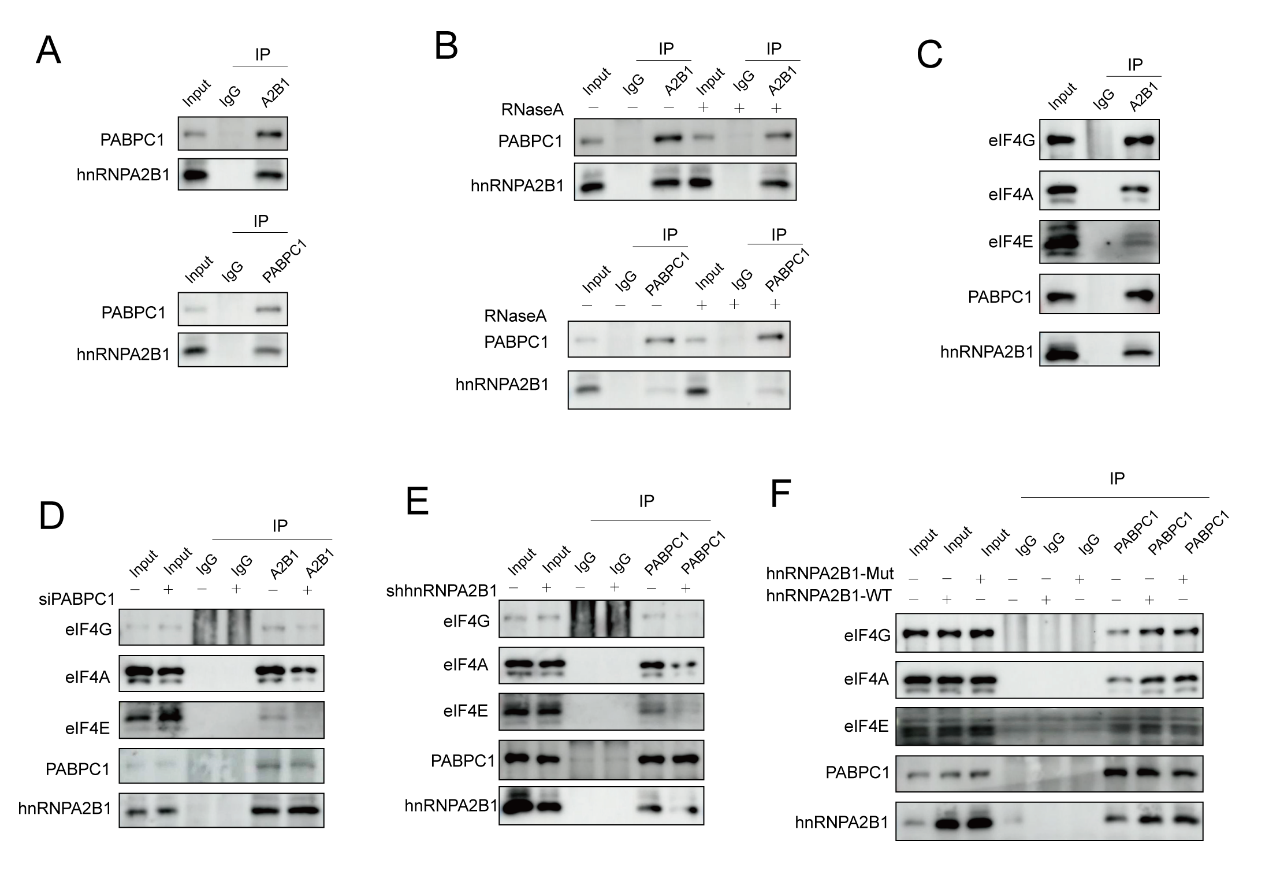


**Figure S6.** hnRNPA2B1 interacted with PABPC1 to promote the mRNA circularization and non-m^6^A translation initiation in AGS cells. (A) hnRNPA2B1 Co-IP with PABPC1 (up) and PABPC1 Co-IP with hnRNPA2B1 (down) in AGS cells. (B) hnRNPA2B1 Co-IP with PABPC1 (up) and PABPC1 Co-IP with hnRNPA2B1 (down) in AGS cells with or without RNase A treatment. (C) hnRNPA2B1 Co-IP with PABPC1 and eIF4F complex. (D) hnRNPA2B1 Co-IP with PABPC1 and eIF4F complex in AGS cells, either with or without si-PABPC1 treatment. (E) PABPC1 Co-IP with hnRNPA2B1 and eIF4F complex in AGS-NC and AGS-shhnRNPA2B1 cells. (F) PABPC1 Co-IP with hnRNPA2B1 and eIF4F complex in hnRNPA2B1-WT or hnRNPA2B1-Mut transduced hnRNPA2B1 knockdown AGS cells.

**Supplementary Tables**

**Table S1 Antibodies used in the experiments**

| Antibodies | Sources | Cat No. |
| --- | --- | --- |
| anti-β-actin | Proteintech | 60008-1-Ig |
| hnRNPA2B1 | Abcam, Proteintech | ab183654 |
| CagA | Santa Cruz Biotechnology | sc-28368 |
| NF-κB | CST | 8242T |
| Phospho-NF-κB p65 (Ser536) | Santa Cruz Biotechnology | sc-136548 |
| PABPC1 | Proteintech | 10970-1-AP，66809-1-Ig |
| anti-eIF4G | CST | #2469 |
| anti-eIF4A | CST | #2013 |
| anti-eIF4E | CST | #2067 |
| Goat Anti-Rabbit IgG (H+L) | Jackson ImmunoResearch | 111-001-003 |
| goat anti-rabbit IgG H&L conjugated to Alexa Fluor 594 | Abcam | ab150080 |
| goat anti-mouse IgG H&L tagged with CoraLite488 | Abcam | ab150113 |
| GPX1 | Proteintech | 29329-1-AP |
| DLAT | Proteintech | 13426-1-AP |
| CIP2A | CST | 14805S |

**Table S2 Primer sequences for RT-qPCR**

| Name | Forward Primer | Forward Primer |
| --- | --- | --- |
| hnRNPA2B1 | AACAGTTCCGTAAGCTCTTTATTGG | TTTTGCTTGCAGGATCCCTCATTAC |
| GAPDH | CTCCTCCTGTTCGACAGTCAGC | CCCAATACGACCAAATCCGTT |
| hnRNPA2B1-P1 | GGGTCATTGCGGCGTGAAC | CCAACTCGGCGGATTGACTC |
| hnRNPA2B1-P2 | AGAGCCTTCCCGCCATTGG | AGAGCCTTCCCGCCATTGG |
| GPX1 | AGAGCCTTCCCGCCATTGG | AGGTGTTCCTCCCTCGTAGGTTTAG |
| DLAT | TGTCCTTCTACTCCAGCATCGTCTC | TGTCCTTCTACTCCAGCATCGTCTC |
| CIP2A | CGCCGCTTCCAGACCATTGAC | AGGTGTTCCTCCCTCGTAGGTTTAG |

**Table S3 The sequences of siRNA or shRNA**

| Name | Sequence |
| --- | --- |
| shhnRNPA2B1-1 | Top strand:  GATCCGCAGAAGAAAGTTTGAGGAACTACTATTCAAGAGATAGTAGTTCCTCAAACTTTCTTCTGTTTTTTG  Bottom strand:  AATTCAAAAAACAGAAGAAAGTTTGAGGAACTACTATCTCTTGAATAGTAGTTCCTCAAACTTTCTTC GCG |
| shhnRNPA2B1-2 | Top strand:  GATCCGCATGGCTGCAAGACCTCATTCAATTTTCAAGAGAAATTGAATGAGGTCTTGCAGCCATGTTT  TTTG  Bottom strand:  AATTCAAAAAACATGGCTGCAAGACCTCATTCAATTTCTCTTGAAAATTGAATGAGGTCTTGCAGCCATGCG |
| siPABPC1-1 | CTAGCCAAATTGCTCAACT |
| siPABPC1-2 | GACGATTTAAGTCTCGTAA |
